# The potential capability of substituting chemical fertilizers with crop straw and human-livestock-poultry manure in areas with different topographic characteristics

**DOI:** 10.1016/j.heliyon.2023.e18845

**Published:** 2023-08-01

**Authors:** Lei Chen, Jingyu Zhang, Xiaolin Xia, Zhi Yang, Bangwen Wang, Changyu Long

**Affiliations:** a Key Laboratory of Water Conservancy and Water Resources in Anhui Province, Heifei 230088, China; b Anhui and Huaihe River Institute of Hydraulic Research, Heifei 230088, China

**Keywords:** Straw and manure, Quantity and composition, Structural differences, Nutrient contents, Potential for alternative fertilizers, Topography

## Abstract

Agricultural production and lifestyle are constrained by topography, causing notable under disparities in the composition, distribution, and environmental benefits of crop straw and human-livestock-poultry manure (CSHLPM) in areas with different topographic characteristics. Under the premise of the international consensus on sustainable development, it has become an objective requirement to improve the use of CSHLPM as fertilizer, and minimize the use of chemical fertilizers. Thus, an investigation was conducted to assess the difference in the quantity and composition of CSHLPM in plain, hill, and mountainous areas, taking Anhui Province of China as a case study, to evaluate the potential contribution of CSHLPM to farmland and identify the environmental benefits. The results show that the composition of CSHLPM in plain, hill, and mountainous areas varied greatly, and the reuse of CSHLPM in farmland could supplement, but not completely replace the use of chemical fertilizer to meet the needs of nutrients. In plain areas, CSHLPM did not provide the necessary amount of N and P_2_O_5_ for crop growth, whereas in mountainous areas, P_2_O_5_ was not available in adequate amounts for crop growth. Only in hilly areas can CSHLPM be used to completely replace chemical fertilizers and supply the necessary amounts of nutrients for crop growth. Based on the characteristics of different topographies, two constructive suggestions for improving the management strategy of CSHLPM were put forward: adjust the industrial structure of husbandry and planting, and specify the recommended ratios for mixing organic chemical fertilizers.

## 1 Introduction

Crop straw and human-livestock-poultry manure (CSHLPM) are the main waste types used in agricultural production activities. During the development of traditional agriculture for thousands of years, our ancestors also realized the value of CSHLPM as a fertilizer resource [[1], [2], [3]]. CSHLPM is rich in nutrients, such as N, P_2_O_5_ and K_2_O, which are necessary for the growth of various types of crops. In actual agricultural production, CSHLPM is often used as an organic fertilizer to improve crop yield [4]. From the perspective of renewable resource recycling, after CSHLPM is returned to the soil, the residues can be used again as a natural fertilizer by crops through the humification and decomposition processes. The use of CSHLPM can improve the soil structure and microecological environment, greatly improving soil fertility [5]. Thus, the use of CSHLPM as a fertilizer plays an active role in both energy resource development and food security strategies [6]. The reutilization of CSHLPM as a farmland fertilizer can reduce the environmental damage and treatment costs caused by CSHLPM itself. Moreover, it can reduce the demand for inorganic fertilizers of N, P_2_O_5_ and K_2_O in the agricultural system and alleviate agricultural nonpoint pollution. The use of CSHLPM is in line with the sustainable development and green agriculture strategy recommended by the international community [7,8]. Therefore, in recent years, with the increasing awareness of resource reutilization and environmental protection, researchers and policy-makers have paid great attention to the issue of reutilizing CSHLPM as a farmland fertilizer.

However, with the wide use of inorganic fertilizers, the utilization ratio of CSHLPM and other organic fertilizers has been significantly reduced due to factors such as reutilization efficiency, economic cost, and ease of use [9]. A large amount of straw is incinerated, discarded and landfilled, and manure is discharged into surface water without sufficient treatment processes. Taking China as an example, in the 1950s, nutrients from organic manures such as crop straw and livestock manure supplied more than 90% of the total nutrients in China's farmland, while the proportion of inorganic fertilizers was extremely low [10,11]. Since the beginning of the 21st century, China has become the largest producer and consumer of inorganic synthetic fertilizers in the world, accounting for approximately 35% of the global total consumption [12]. The vast majority of China's farmland nutrient demand is provided by chemical fertilizers [13]. The reduction in the CSHLPM utilization ratio leads to an inadequate linkage between the husbandry system and planting system [14]. It is difficult for CSHLPM nutrients to be used as agricultural fertilizer and its use poses high environmental risks (i.e., agricultural nonpoint pollution) to the ecosystem by discharging CSHLPM into the environment without proper treatment [15]. Under the premise of the international consensus on sustainable development, it has become an objective requirement to reduce environmental risks, improve the reutilization of CSHLPM as fertilizer, and minimize the use of chemical fertilizers [16]. A comprehensive understanding of the composition, distribution characteristics, and potential of CSHLPM as an alternative fertilizers is the precondition to reaching this goal [11,17].

Topography is an important factor affecting the distribution of agricultural production [18], and it affects the distribution of agricultural crops by influencing the redistribution of crop production conditions such as light, heat, water, soil, and fertility [19,20]. Plain, hill, and mountainous areas of the same size often have different available land areas, limiting the associated agricultural production and lifestyle [[21], [22], [23]], resulting in significant differences in livestock breeding scale and crop planting types. This leads to significant differences in the composition and distribution characteristics of CSHLPM in areas with different topographic characteristics, and further affects the potential of alternative fertilizers and the environmental benefits of using CSHLPM. Meanwhile, policy-makers are challenged by some new dilemmas. First of all, with the development of industrialization in recent decades, livestock and poultry breeding industries are changing in structure toward being larger, more commercial, and more intensive [24,25]. Accordingly, the emission levels and pollution risks of livestock manure are more concentrated and magnified [26]. Second, it has become a consensus that urbanization will lead to a sharp decrease in cultivated land area, but the change range of cultivated land area in different regions is not consistent [27]. More seriously, on the premise of an inadequate linkage between the husbandry system and planting system, livestock and poultry breeding and crop production are independent of each other. There is an obvious interregional decoupling between the quantity of CSHLPM and the nutrient demand of cultivated land [10,11]. A practical choice for the management strategy of the sustainable utilization of CSHLPM must carry out targeted analysis and treatment on this interregional decoupling phenomenon.

In this study, an investigation was conducted to assess the difference in the quantity and composition of CSHLPM in plain, hill, and mountainous areas, taking Anhui Province of eastern China as a typical research case (the area is characterized by a topography consisting of plains, hills and mountains in a ratio of approximately 1:1:1), to evaluate the potential contribution of CSHLPM to farmland and its environmental benefits. First, the differences in the quantity and composition of CSHLPM in the various topographic areas were evaluated ascertain the nutrient content of CSHLPM and provide comprehensive basic data. Additionally, the potential contribution of CSHLPM in areas with different topographic characteristics to agricultural and environmental benefits was assessed by comparing it to the nutrient requirements of crops in historical periods. Finally, recommendations were made regarding the management of CSHLPM.

## 2 Materials and methodology

### 2.1 Study regions and original database resources

**Fig. 1:**
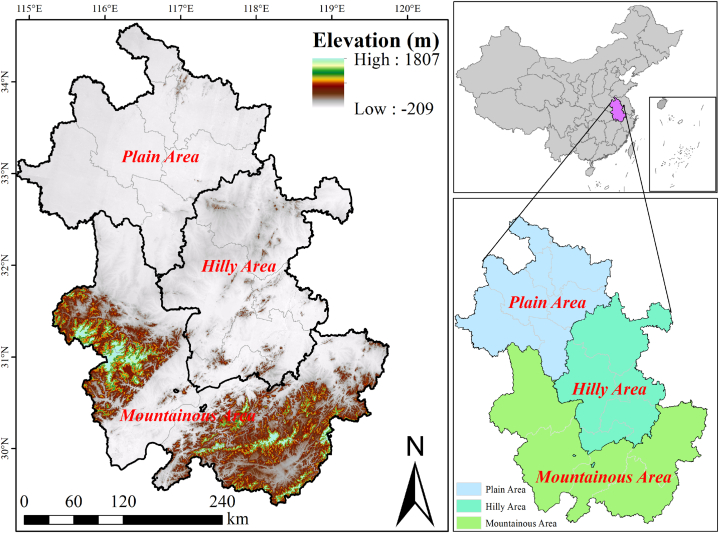
The general features of topography in the study area.

Anhui, located in the Yangtze River Delta region of eastern China, covers an area of 140,100 square kilometers. It is situated between 114° 54′ and 119° 37′ E and 29° 41′ and 34° 38′ N, with an average annual temperature of 14–17 °C and precipitation of 770–1700 mm. The Yangtze River and Huai River traverse the entire region from west to east. The topography of this area is dominated by plains, hills and low mountains, which appear gradually from north to south, with plains, hills, and mountains accounting for 31.3%, 29.5%, and 31.2% of the total area of the province, respectively (Fig. 1). This region is endowed with abundant light, heat, and water resources, making it an ideal agricultural production base in eastern China that is suitable for both agricultural and animal husbandry production.

Data for this study, such as population, crop planting, and livestock and poultry breeding data, were sourced from regional statistical yearbooks (http://tjj.ah.gov.cn/), statistical yearbooks of cities, and other published scientific literature. The evaluation was conducted in 16 municipalities of Anhui Province, which were divided into three regions as follows: the plain area in the north, comprising Bengbu, Huaibei, Huainan, Fuyang, Suzhou and Bozhou; the hilly area in the middle, between the Yangtze River and Huai River, including Hefei, Wuhu, Maanshan, Tongling, Chuzhou; and the mountainous area in the west and south, encompassing Anqing, Huangshan, Lu'an, Chizhou, and Xuancheng.

### 2.2 Yield of crop and straw

Anhui Province is a major agricultural production base in eastern China, with a large amount of crop straw produced annually. This area cultivates a variety of crops, such as rice, wheat, maize, soybean, potato, peanut, canola, cotton, sesame, sugarcane, sugar beet, and tobacco. However, this study focused on the five most important crops: rice, wheat, corn, soybean, and potato, which made up 82.8% of the total agricultural cultivation area in 2020. The sown areas of the 5 main crops in Anhui Province from 2011 to 2020 ranged from 6.57 to 7.28 million hectares (Table S1), and the grain output of the 5 main crops during 2011–2020 varied from 35.58 to 40.1 million tons (Table S2).

Straw yield is a measure of the residues or byproducts of harvesting crops, which are composed of stalks, stubbles, and leaves. Residue-to-production ratios (RPRs), as a systemic evaluation index, have widely been used to determine straw yield [28]. Straw yield can be calculated by using Eq. (1):

(1) $$SY_{sum}=\sum_{i=1}^{n}SY_{i}=\sum_{i=1}^{n}WP\times SG$$

where *SY*_*sum*_ is the theoretical sum of straw yield in a year, *WP* is the coefficient of residue-to-production ratios showed in Table 1, *SG* is the grain output in a year, and *i* is the crop type.

**Table 1:** The RPRs of different crops used in this study.

Crops	Rice	Wheat	Corn	Soybean	Potato
RPRs	1	1.1	2	1.6	0.5

### 2.3 Output of human-livestock-poultry manure

The theoretical output of human-livestock-poultry manure can be calculated by the excretion coefficient method [29,30]. Feces and urine yields were calculated separately by partitioning different excretion coefficients of excrement of humans, livestock, and poultry. The excretion coefficient depends on several variables, e.g. regional environment, animal species, and feeding mode. Table 2 lists the adopted emission coefficients and breeding periods according to the relevant literature [11,14]. The feces and urine outputs of humans were calculated based on the number of permanent residents in a given rural area, while the output of livestock and poultry needs to consider slaughters for poultry and pigs in addition to year-end inventory for livestock. For poultry and pigs, we counted the actual number of actual breeding days, and the growth period of other livestock was considered longer than one year. In the process of numerical calculation, the value for pig, poultry, and other livestock is commonly set as 199, 210 and 365 days, respectively. The formulas for calculation are as follows (Eq. (2) and Eq. (3)):

(2) $$P_{1}=R\times q\times 365$$

(3) $$P_{j}=M_{j}\times Q_{j}\times T_{j}$$

where *P*_*1*_ denotes the annual excreta production of humans; *R* is the number of permanent rural residents; *q* is the excretion coefficient of feces or urine; *P*_*j*_ denotes the annual output of livestock and poultry breeding for animal type *j*; *M*_*j*_ is the number of livestock and poultry; *Q*_*j*_ is the daily output for animal type *j*; and *T*_*j*_ is the breeding period for animal type *j*. The studied livestock and poultry included cattle, sheep, pig, chicken, and duck, which cover the main varieties of livestock and poultry breeding in this region. *T*_*j*_ was arbitrarily capped at 365 d for animals that required more than 1 year to reach maturity.

**Table 2:** Estimated coefficients of waste production from livestock, poultry and humans.

Pollutant Sources	Coefficient of pollutant emission unit: kg/(d✕head)		Breeding periods unit: d
	Feces	Urine	
Human	0.15	2	365
Cattle	20	10	365
Sheep	2.6	0.64	365
Poultry	0.12	–	210
Pig	2	3.3	199

### 2.4 Nutrient resources of CSHLPM

Essential nutrients (EN) derived from CSHLPM are commonly indicated by the net quantity of N, P_2_O_5_ and K_2_O. We calculated the *EN* amount for each crop type and livestock-poultry type. Thus, *EN*_*Nsum*_, *EN*_*Psum*_ and *EN*_*Ksum*_ can be illustrated by the following mathematical calculation models [13] as follows (Eqs. ((4), (5), (6))):

(4) $$EN_{Nsum}=\sum_{i=1}^{n}SY_{i}\times C_{Ni}+\sum_{j=1}^{m}P_{j}\times D_{Nj}$$

(5) $$EN_{Psum}=\left(\sum SY_{i}\times C_{Pi}+\sum P_{i}\times D_{Pi}\right)\times 2.29$$

(6) $$EN_{Ksum}=\left(\sum SY_{i}\times C_{Ki}+\sum P_{i}\times D_{Ki}\right)\times 1.2$$

where *EN*_*Nsum*_, *EN*_*Psum*_ and *EN*_*Ksum*_ are the total nutrient contents derived from the CSHLPM of N, P_2_O_5_ and K_2_O, respectively. *C*_*N*_, *C*_*P*_, and *C*_*K*_ are the nutrient content coefficients of N, P and K, respectively, derived from crop straw. Similarly, *D*_*N*_, *D*_*P*_, and *D*_*K*_ are the coefficients of N, P and K obtained from livestock-poultry manure. Additionally, two conversion coefficients (2.29 and 1.2) indicate the ratios of P_2_O_5_ and K_2_O to P and K. Table 3 lists the total nutrient contents of N, P_2_O_5_ and K_2_O in different types of straw and animal manure.

**Table 3:** N, P_2_O_5_ and K_2_O contents in different types of straw and animal manure (wt% of fresh excrement) [13,31].

Pollutant Sources		N (%)	P_2_O_5_ (%)	K_2_O (%)
Straw	**Rice**	0.91	0.30	2.27
	**Wheat**	0.65	0.18	1.26
	**Corn**	0.92	0.35	1.42
	**Soybean**	1.81	0.46	1.40
	**Potato**	2.37	0.65	3.66
Human	**Feces**	1	0.5	0.37
	**Urine**	0.5	0.13	0.19
Cattle	**Feces**	0.38	0.1	0.24
	**Urine**	0.51	0.02	0.92
Sheep	**Feces**	1.01	0.22	0.54
	**Urine**	0.7	0.03	0.77
Poultry	**Feces**	0.81	0.37	0.62
	**Urine**	–	–	–
Pig	**Feces**	0.55	0.26	0.3
	**Urine**	0.18	0.02	0.16

### 2.5 Potential capability of substituting CSHLPM for chemical fertilizer

By incorporating straw and manure into the soil, the need for chemical fertilizers can be diminished, as the nutrients they provide can be beneficial in sustaining the fertility of the soil. The N, P_2_O_5_ and K_2_O nutrient requirements of various crops, coupled with the outputs of such crops, can be used to calculate the plant nutrient demand, as shown in Eq. (7).

(7) $$F_{Nsum}\left(F_{Psum}\;\text{or}\;F_{Ksum}\right)=\sum_{i=1}^{n}P_{i}\times QN_{i}\left(QP_{i}\;\text{or}\;QK_{i}\right)\times 10^{-2}$$

where *F*_*Nsum*_, *F*_*Psum*_ and *F*_*Ksum*_ are the total nutrient demand for crop growth, *P*_*i*_ is the total output of crop *i*, and *Q*_*i*_ is the N, P_2_O_5_ and K_2_O demand per 100 kg output of crop *i*, as shown in Table 4.

**Table 4:** Amount of nutrients to be absorbed by crops per unit yield (kg/100 kg).

Crops	N	P_2_O_5_	K_2_O
Rice	1.46	0.62	1.92
Wheat	2.46	0.85	2.77
Corn	2.58	0.98	2.78
Soybean	8.14	2.30	3.20
potato	0.53	0.14	0.60

To determine the extent to which CSHLPM can potentially substitute the nutrients needed by crops, the average nutrients of CSHLPM were compared to the nutrients required by crops. Specifically, the following Eq. (8) was calculated for the comparison:

(8) $$E_{\left(N,P,K\right)}=\frac{EN_{\left(N,P,K\right)}}{F_{\left(N,P,K\right)}}$$

where *EN*_*(N,P,K)*_ represent the net quantities of N, P_2_O_5_ and K_2_O in CSHLPM, respectively. *F*_*(N,P,K)*_ represents the N, P_2_O_5_ and K_2_O in the nutrients needed by crop growth, respectively. *E*_*(N,P,K)*_ represents the percentages of N, P_2_O_5_ and K_2_O that CSHLPM can supply in place of nutrients, respectively.

In this paper, considering China's current sustainable energy utilization and future development planning [13,32], we assumed that the return rate of CSHLPM ranged from 0.5 to 1, and the release rate of nitrogen, phosphorus, and potassium nutrients was 100%. Additionally, the remaining nutrients needed for crop growth were supplemented by a compound fertilizer with a ratio of N, P_2_O_5_ and K_2_O of 1:1:0.8 [31,33]. Thus, the compound fertilizer demand was calculated as follows (Eqs. ((9), (10), (11))):

(9) $$NS_{\left(N,P,K\right)}=F_{\left(N,P,K\right)}-EN_{\left(N,P,K\right)}$$

(10) $$CS_{\left(N,P,K\right)}=\left\{ \begin{array}{cc} \frac{NS_{\left(N,P,K\right)}}{R_{\left(N,P,K\right)}} & NS_{\left(N,P,K\right)}>0\\ 0 & NS_{\left(N,P,K\right)}\leq 0 \end{array}\right.$$

(11) $$CS_{\max}=\mathrm{MAX}\left(CS_{N},CS_{P},CS_{K}\right)$$

where *NS*_*(N,P,K)*_ represents the net respective quantities of nutrients that need to be supplemented with compound fertilizer, respectively. *R*_*(N,P,K)*_ represents the respective conversion ratio of nutrients to compound fertilizer. And *CS*_*max*_ refers to the amount of compound fertilizer required when all nutrient requirements are met.

## 3 Results

### 3.1 Quantity and composition of crop straw

**Fig. 2:**
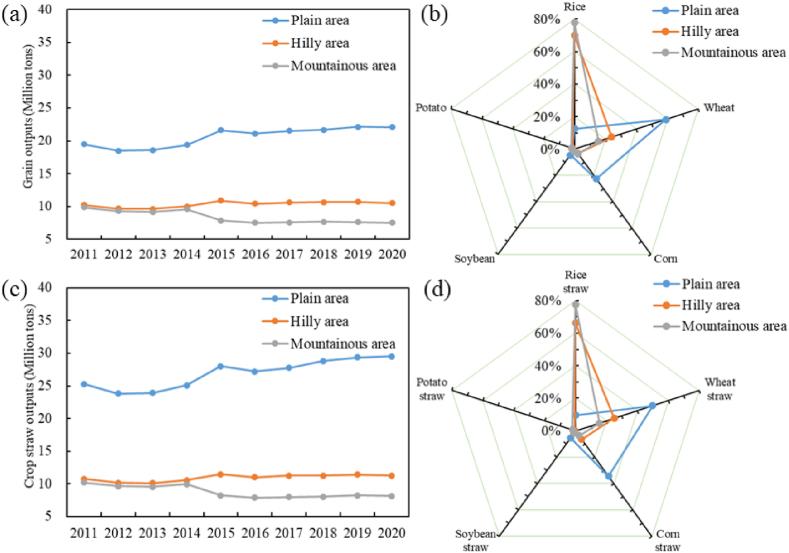
(a) Annual change in grain output (million tons) of the 5 main crops (rice, wheat, corn, soybean, and potato) in the three topographic areas (plain, hilly, and mountainous areas), (b) structural variation in average grain output from 2011 to 2020, (c) annual change in crop straw output, and (d) structural variation in average crop straw output from 2011 to 2020.

Fig. 2 (a) presents the grain output (million tons) in the three topographic regions from 2011 to 2020. It is evident that the plain area had the highest grain output, with an annual average of 20.61 million tons, while the hilly and mountainous areas had 10.34 and 8.38 million tons respectively. Furthermore, the temporal variation in the grain output in these three areas showed a different trend; it has increased slightly in the plain area, remained relatively stable in the hilly area, and decreased slightly in the mountainous area. Fig. 2 (b) demonstrates that grain output structures in the three topographic areas varied drastically due to topography, climate, and other factors. Wheat and corn dominated the plain area, comprising 59.1% and 22.6% respectively, whereas the other two areas were planted mainly with rice; the mountainous area had an even higher proportion of rice, at nearly 80%.

An entirely different grain output structure and significantly different RPRs among crop expansions have caused observed the regional gap in the total amount of crop straw, as illustrated in Fig. 2 (c). Among the three regions, the plain area was the highest producer of crop straws, followed by the hilly area and the mountainous area. Rice, wheat, and corn were the predominant sources of crop straw, comprising 94.1%, 97.6%, and 96.8% of the total crop straw output in the plain, hilly, and mountainous areas, respectively. Nevertheless, similar to the structure of grain outputs, the structure of crop straw among the three topographic areas was significantly different. In a comparison of Fig. 2 (b) and Fig. 2 (d), the amount of crop straw in the plain area with high corn production has increased considerably due to the higher RPR of corn than that of rice and wheat. The contribution of corn straw to the total straw increased to 34.6%, which was higher than the contribution (22.6%) of corn output to the total grain output. The contribution of the three main crop straws in the plain area was in the order of wheat (49.9%), corn (34.6%) and rice (9.6%), while in the hilly area and mountain area, the order was rice (66.2% and 74.0%), wheat (25.0% and 16.0%) and corn (6.4% and 6.8%), respectively.

### 3.2 Quantity and composition of human-livestock-poultry manure

Statistical data from 2011 to 2020 revealed a substantial disparity in the amount and composition of human-livestock-poultry manure in areas with different topographic characteristics. The statistical data are summarized in the Supplementary Material (Table S3). The variations in the amount of livestock, poultry, and humans certainly caused a shift in the amount of human-livestock-poultry manure, in terms of both feces and urine. Table 5 shows that the production of human-livestock-poultry's feces and urine varied from 2011 to 2020 in the three topographic areas, with the highest quantity in the plain area, followed by the hilly area and the mountainous area. The quantity of human-livestock-poultry manure in the three topographic regions decreased from 2011 to 2020, with a peak value between 2013 and 2015. The quantity of feces decreased from 24.20 Mt (plain area), 15.12 Mt (hilly area), and 7.13 Mt (mountainous area) in 2011 to 21.62 Mt, 8.68 Mt, and 5.74 Mt in 2020, representing a reduction rates of 10.7%, 42.6% and 19.6%, respectively. Similarly, the quantity of urine decreased from 31.04 Mt, 20.69 Mt, and 11.15 Mt in 2011 to 27.18 Mt, 12.25 Mt, and 7.45 Mt in 2020, with a reduction rates of 12.4%, 40.8% and 33.1%, respectively.

**Table 5:** Quantity of human-livestock-poultry manure among the three topographic areas. Unit: million tons.

Area	Wastes	2011	2012	2013	2014	2015	2016	2017	2018	2019	2020
Plain area	**Feces**	24.20	25.31	26.22	26.86	29.31	29.76	30.07	20.09	20.03	21.62
	**Urine**	31.04	31.68	32.11	32.63	34.52	34.42	34.42	27.68	26.37	27.18
Hilly area	**Feces**	15.12	15.70	15.87	16.11	14.46	14.13	13.99	9.72	8.96	8.68
	**Urine**	20.69	20.85	20.92	21.04	19.13	18.69	18.47	16.23	14.18	12.25
Mountainous area	**Feces**	7.13	7.35	7.61	7.58	7.67	7.47	7.40	5.56	5.48	5.74
	**Urine**	11.15	11.25	11.26	11.24	11.18	10.90	10.75	9.31	8.21	7.45

**Fig. 3:**
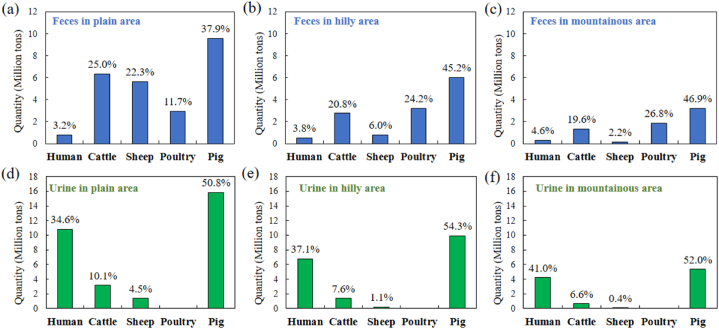
Quantity and composition of the average human-livestock-poultry manure from 2011 to 2020.

Meanwhile, over the last 10 years, the composition of human-livestock-poultry manure among the three topographic areas was examined (as shown in Fig. 3), and it was evident that the composition, content, and ratio of human-livestock-poultry manure varied significantly. In the plain area, statistics showed that the main sources of human-livestock-poultry manure were pigs (9.61 Mt), cattle (6.33 Mt) and sheep (5.64 Mt), which together accounted for 37.9%, 25% and 22.3% of the total, respectively (Fig. 3(a)). In the hilly area, the main sources of human-livestock-poultry manure were pigs (6.00 Mt), poultry (3.21 Mt) and cattle (2.76 Mt), making up 45.2%, 24.2% and 20.8% of the total, respectively (Fig. 3(b)). The same was true for the mountainous area, where pigs, poultry and cattle accounted for 46.9%, 26.8% and 19.6% of the total, respectively (Fig. 3(c)). The composition characteristics of human, livestock, and poultry manure in plain, hilly, and mountainous areas differed in the proportion of sheep and poultry manure production. For instance, in the plain area, sheep and poultry feces accounted for 22.3% and 11.7% of the total feces, respectively, while in the hilly and mountainous areas, these proportions were 6.0%, 24.2% and 2.2%, 26.8%. Notably, in comparison to other sources, pigs produced the largest quantities and proportions of feces and urine, with the percentage of pig urine reaching up to 50% of the total urine (Fig. 3(d–f)). This phenomenon can be attributed to the difference in livestock and poultry breeding structure in areas with different topographic characteristics, which is affected by the amount of available land and geographical environment, given that the residents' diet structure is mainly centered around pork. The plain area, with its flat terrain and more available land, is suitable for the grazing and breeding needs of large livestock such as cattle and sheep, while the mountainous and hilly areas, with their limited available land, are more suitable for intensive poultry breeding.

### 3.3 Nutrients in CSHLPM

The composition and quantity of agricultural and animal husbandry waste varied greatly among the different topographical areas. The nutrient content (N, P_2_O_5_ and K_2_O) of these wastes was closely linked to the types of crop straw, livestock, and poultry species present. The difference in the quantity and composition of the agricultural and animal husbandry wastes in different regions, as well as the difference in nutrient content in different wastes, resulted in a significant difference in nutrients across the different regions. First, with respect to regional distribution, the nutrients in the CSHLPM in Anhui Province were concentrated mainly in the northern plain region and central hilly area, with a stark contrast in the amount of nutrients in different regions. Table 6 demonstrates that the nutrient resources produced in the three topographic areas ranged from 6.4 to 61.2 ( × 10^4^ t), with average N, P_2_O_5_ and K_2_O nutrients of 50.0, 14.9, and 56.6 ( × 10^4^ t) in the plain area, 23.8, 7.6, and 30.7 ( × 10^4^ t) in the hilly area, and 15.6, 5.1, and 22.9 ( × 10^4^ t) in the mountainous area respectively. Additionally, the source and distribution of nutrients from the CSHLPM were significantly disparate.

**Table 6:** Nutrients in the CSHLPM of the three topographic areas from 2011 to 2020. Unit: 10^4^ t.

Areas	Nutrient	2011	2012	2013	2014	2015	2016	2017	2018	2019	2020
Plain area	**N**	48.5	47.8	48.4	49.7	54.5	54.0	54.6	46.9	47.2	48.5
	**P**_**2**_**O**_**5**_	14.3	14.0	14.2	14.7	16.2	16.0	16.2	14.4	14.5	14.8
	**K**_**2**_**O**	53.7	52.1	52.7	54.6	61.2	60.2	61.2	56.0	56.7	57.7
Hilly area	**N**	25.7	25.5	25.5	26.0	25.2	24.5	24.5	21.0	20.4	19.5
	**P**_**2**_**O**_**5**_	8.1	8.1	8.1	8.2	8.1	7.9	7.9	6.8	6.6	6.3
	**K**_**2**_**O**	32.1	31.2	31.0	32.0	32.7	31.5	31.8	28.9	28.5	27.7
Mountainous area	**N**	17.2	16.9	16.9	17.3	15.9	15.4	15.4	13.8	13.9	13.6
	**P**_**2**_**O**_**5**_	5.5	5.4	5.5	5.6	5.2	5.0	5.0	4.5	4.6	4.5
	**K**_**2**_**O**	26.2	25.1	24.9	25.7	22.4	21.5	21.6	20.6	20.6	20.4

**Fig. 4:**
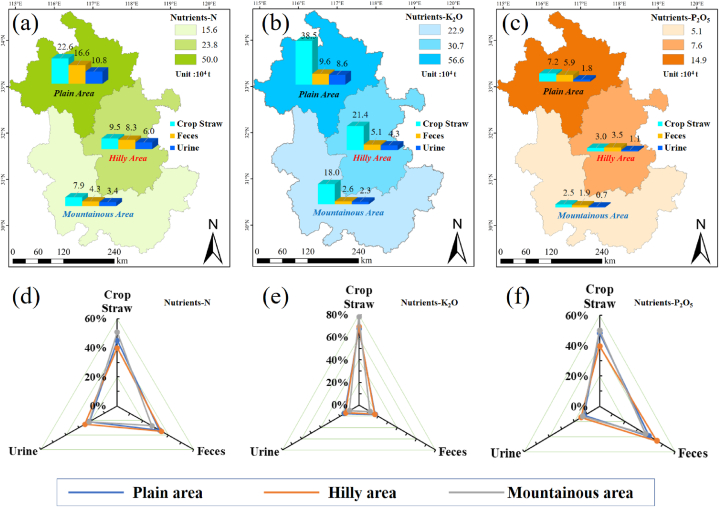
Quantity, distribution and composition of N, P_2_O_5_ and K_2_O, as well as total nutrients, in CSHLPM of the three topographic areas.

Fig. 4 illustrates the average N, P_2_O_5_, and K_2_O as well as total nutrients from 2011 to 2020 in the CSHLPM across the three topographic areas. It is evident that the sources of various nutrient types are distinct due to the varying nutrient content of the wastes (Fig. 4(a–c)). Irrespective of the kind of nutrients, crop straw in mountainous areas accounted for higher proportions (N-50.8%, P_2_O_5_-50.0% and K_2_O-78.4%) than that in plain areas (N-45.2%, P_2_O_5_-48.4% and K_2_O-67.9%) and hilly area (N-40.0%, P_2_O_5_-39.9% and K_2_O-69.6%) (as shown in Fig. 4(d–f)). The proportion of nutrients-K_2_O from crop straw was the highest, approximately 70%, while the proportion of nutrients-N and nutrients-P_2_O_5_ from crop straw was only 45.3% and 46.2%, respectively, which was lower than that of feces and urine.

## 4 Discussion and policy suggestions

### 4.1 Supplement or replacement of chemical fertilizer by CSHLPM

CSHLPM is frequently employed as an organic fertilizer in agricultural production to enhance crop yield, with the majority of it (over 50%) being returned to farmland after undergoing treatment [31]. Due to the encouragement from both the public and the government, as well as the global acknowledgement of environmental protection and sustainable development, CSHLPM is increasingly returning to farmland for reuse. The residues of CSHLPM can be recycled as natural fertilizer for crops due to the humification and decomposition processes that occur when it is returned to the soil. This process enhances the soil structure and microecological environment, consequently increasing soil fertility and reducing environmental pollution during agricultural production. To enhance the utilization efficiency of CSHLPM, it is essential to gain an in-depth understanding of the extent to which it can substitute the use of fertilizers. By comparing the nutrients needed for crop growth with those derived from CSHLPM, assuming that nutrients can be completely incorporated into soils after returning to the fields, the potential of CSHLPM can be evaluated.

In view of the considerable disparity in the amount of cultivated land and crop planting types, the total nutrient necessities in the three topographic areas also demonstrated a remarkable divergence. Table 7 provides a breakdown of the nutrient requirements of crop growth. On average, the total nutrient requirements in the plain area amounted to 126.63 × 10^4^ t/a in 2011–2020, which included 53.28 × 10^4^ t of N, 18.64 × 10^4^ t of P_2_O_5_, and 54.71 × 10^4^ t of K_2_O. However, the average total nutrient requirements in hilly and mountainous areas were 48.03 × 10^4^t (N: 18.59 × 10^4^ t, P_2_O_5_: 7.23 × 10^4^ t, and K_2_O: 22.21 × 10^4^ t) and 27.60 × 10^4^ t (N: 14.51 × 10^4^ t, P_2_O_5_: 5.72 × 10^4^ t and K_2_O: 17.37 × 10^4^ t), respectively.

**Table 7:** Quantity of nutrients required for crop growth in the three topographic areas from 2011 to 2020. Unit: 10^4^ t.

Areas	Nutrient	2011	2012	2013	2014	2015	2016	2017	2018	2019	2020
Plain area	**N**	52.2	49.3	49.2	50.8	55.2	53.5	54.5	55.4	56.3	56.4
	**P**_**2**_**O**_**5**_	18.0	17.0	17.0	17.6	19.4	18.8	19.2	19.6	19.9	19.9
	**K**_**2**_**O**	51.6	49.2	49.5	51.7	57.1	55.7	56.8	57.6	58.9	59.0
Hilly area	**N**	18.2	17.3	17.2	17.9	19.3	18.7	19.1	19.3	19.6	19.4
	**P**_**2**_**O**_**5**_	7.1	6.7	6.7	7.0	7.5	7.3	7.4	7.5	7.6	7.5
	**K**_**2**_**O**	21.8	20.6	20.5	21.4	23.2	22.3	22.7	23.2	23.4	23.0
Mountainous area	**N**	16.8	16.0	15.8	16.5	13.5	13.2	13.2	13.2	13.5	13.4
	**P**_**2**_**O**_**5**_	6.7	6.3	6.2	6.5	5.3	5.2	5.2	5.2	5.3	5.3
	**K**_**2**_**O**	20.3	19.2	19.0	19.8	16.1	15.6	15.6	16.0	16.1	15.9

**Fig. 5:**
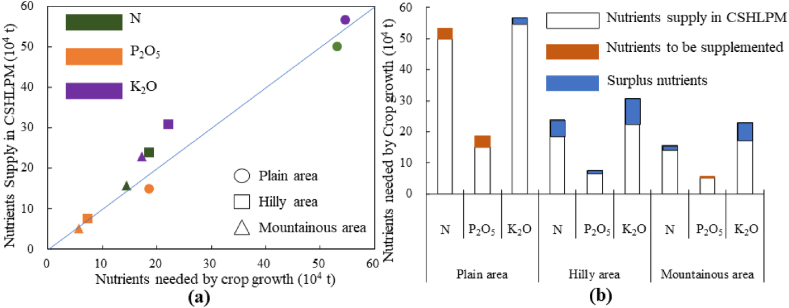
Quantity of nutrients needed for crop growth and those derived from CSHLPM in the three topographic areas. Note: The blue line in panel (a) represents the balance of nutrient supply in CSHLPM and nutrients needed for crop growth.

A thorough comprehension of the regional and species discrepancies of repurposing CSHLPM to satisfy the nutrient requirements of crop growth is essential for improving the efficiency of CSHLPM reuse, thereby reducing the demand for fertilizer. Additionally, the results of this analysis can provide an ideal opportunity for the potential transfer of renewable resources across regions, ultimately leading to an increase in farmer income and a decrease in rural environmental pollution. The results of comparing the nutrients needed for crop growth with those derived from CSHLPM, as shown in Fig. 5, suggest that the reuse of CSHLPM in farmland can be used as a supplement, but cannot completely replace, the use of chemical fertilizer for meeting the needs of nutrients. Moreover, of all the nutrient types, K_2_O was the only one that could be fully satisfied by reusing CSHLPM in all regions, with the amount of K_2_O needed for crop growth being more than that of N and P_2_O_5_ (as shown in Fig. 5(a)). This result implies that, should all CSHLPM resources be returned to the farmlands and be fully reused in the future, all synthetic and costly chemical potassium fertilizers could be eliminated. In addition, the analysis of the suitability of CSHLPM as a fertilizer substitute or supplement reveals that the substitution potential of areas with different topographic characteristics in the studied area varied significantly. In the plain area, the annual average substitution sufficiency of N, P_2_O_5_, and K_2_O was 93.9%, 80%, and 100%, respectively. In the mountainous area, the corresponding values were 100%, 88.6%, and 100% (Fig. 5(b)). Reuse of CSHLPM in the plain area was insufficient to meet the needs of N and P_2_O_5_, while P_2_O_5_ was not adequately supplied in the mountainous area. Notably, the hilly area was the only region in which chemical fertilizer could be completely replaced by CSHLPM, as the annual average substitution sufficiency of N, P_2_O_5_, and K_2_O all reached 100%.

It is evident that the nutrients obtained from CSHLPM are capable of meeting the nutrient requirements of crops, with the calculated nutrient content yielding an average substitution sufficiency of at least 80% for each topographic area for N, P_2_O_5_, and K_2_O. The reuse of CSHLPM is particularly beneficial in alleviating the potassium fertilizer shortage. Nevertheless, the collection, transportation, treatment, processing, and utilization of CSHLPM resources may result in nutrient losses, especially in remote rural areas with poor road infrastructure. Consequently, the rate of CSHLPM return and the rate of nitrogen, phosphorus, and potassium release are essential metrics needed to assess the effectiveness of CSHLPM resource utilization. In this paper, according to China's current sustainable energy utilization and future development plans, we assumed that the return rate of CSHLPM was between 0.5 and 1 and that the release rate of nitrogen, phosphorus, and potassium nutrients was 100%. The remaining nutrients necessary for crop growth were supplemented by compound fertilizers, with the amount of compound fertilizer per unit cultivated land area being utilized as a measure to assess fertilizer demand in different regions.

**Fig. 6:**
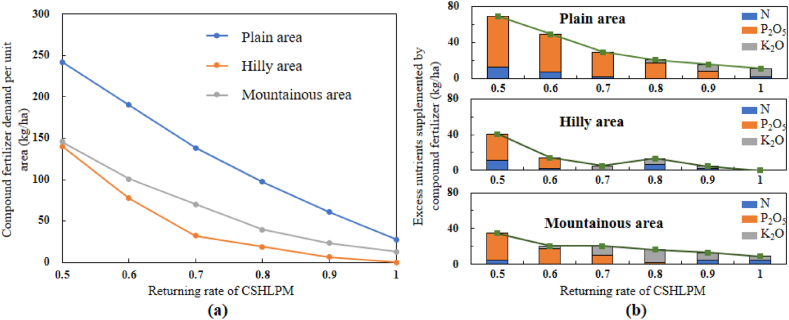
Quantity of compound fertilizer required per unit of cultivated land area by the three topographic areas with different return rates.

Fig. 6 illustrates the fertilizer demand level under different return rates of CSHLPM in areas with different topographic characteristics. The demand for compound fertilizer decreased with the increase in the return rate of CSHLPM, thus addressing the issue of excessive nutrients and its associated environmental problems. Specifically, when the return rate of CSHLPM increased from 0.5 to 1.0, the use of compound fertilizer in plain, hilly, and mountainous areas decreased by 214.8 kg/ha, 140.5 kg/ha, and 132.7 kg/ha (Fig. 6(a)), respectively. The hilly area had the lowest demand for chemical fertilizer under each return rate of CSHLPM, and the demand for fertilizer decreased significantly when the rate of return increased from 0.5 to 0.7. In addition, as shown in Fig. 6(b), while increasing the return rate of CSHLPM was generally an effective method to reduce the overuse of organic fertilizer, a surprising result was observed when the return rate of CSHLPM in hilly areas increased from 0.7 to 0.8, which represented an increase in the overuse of organic fertilizer.

### 4.2 Limitations and uncertainties

This study aimed to investigate the potential contribution of CSHLPM to farmland and identify the environmental benefits by comparing the differences in the quantity and composition of CSHLPM in plain, hilly, and mountainous areas using Anhui Province of China as an example. However, because of the complexity of the research fields, methods and data, this study also faced some existing limitations and assumptions in the process of conducting the research.

First, there are many factors that affect the nutrient supply by CSHLPM and the nutrient demand by crop growth, but in this paper, not all factors were considered and thus may lead to limits on this research. We focused on the significant differences in crop variety, livestock varieties, and nutrient types among different topographic areas. We have endeavored to use data from reliable sources; however, due to the complexity of the statistical data, we have chosen only the most important content for calculation. For example, we assumed that the source of crop straw was only from five main crops: rice, wheat, corn, soybean, and potato, which account for more than 80% of the total agricultural cultivation area.

Second, in this study, various calculation constants derived from previous research were utilized to calculate residue-to-production ratios and nutrient content [11,13,14,31], which may affect the conclusions of the paper. We understand that these constants may change with space and time. Thus, the selected constants were mainly sourced from recent studies of adjacent areas so that they would likely be more accurate. Simultaneously, it is essential to emphasize that the scope of this research does not extend to the regional applicability of these constants. Instead, the primary aim of this paper was to explore the potential of nutrient substitution with fertilizers.

Third, this paper examined the fertilizer demand under varying return rates of CSHLPM, assuming that all remaining nutrients for crop growth were supplemented by a compound fertilizer with a ratio of N, P_2_O_5_ and K_2_O of 1:1:0.8 [31,33]. However, this hypothesis does not accurately reflect reality, as single-type inorganic fertilizers (N, P, or K) are widely used in some areas, and the proportions of different nutrients in compound fertilizers vary according to regional needs.

### 4.3 Policy suggestions

Since the 1980s, as China's economy has seen drastically changes, its husbandry and agricultural planting industries have also been subject to industrial restructuring. The resources of organic fertilizers, including CSHLPM resources, and the total amount of nitrogen, phosphorus and potassium nutrients returned to farmland have been increased [34,35]. Our research findings indicate that the use of chemical fertilizers can be substantially reduced by fully utilizing these resources, thereby mitigating agricultural nonpoint source pollution, regardless of the topography of the area.

Investigating how to make the return of CSHLPM resources more efficient is the current challenge. Technical issues are not the only factor influencing the return rate, as government policies and industrial structure also play major roles. For example, in the period from the 1980s to the 2010s, China experienced industrialization and a booming livestock breeding industry. This resulted in a decrease in the rate of nutrient return from livestock manure, from 44.88% to 38.61%, while the return of crop straw to farmland was supported by national policies, leading to an increase from 47.92% to 71.27%. To improve the utilization of CSHLPM resources, based on the above research, this paper proposes three constructive management strategies.

**Adjust the industrial structure for husbandry and planting.** By leveraging the natural characteristics and industrial organization of these regions, a judicious modification of the agricultural and horticultural structure can reduce waste and enhance the connection between crop cultivation and husbandry to reduce contamination [36]. For example, the phosphorus content of CSHLPM is insufficient for mountainous areas, so it is advisable to increase the number of livestock and crop species with a high P_2_O_5_ content in their waste products.

**Specify recommended ratios for mixing organic and chemical fertilizers.** Organic fertilizers are not as effective as chemical fertilizers in terms of their immediate effect on crop growth [10]. However, from the perspective of nutrient utilization and environmental governance, it is essential to specify recommended ratios for mixing organic and chemical fertilizers based on the production and recycling of CSHLPM in various regions without compromising agricultural output and farmers' income [37].

## 5 Conclusions

In this paper, an investigation was conducted to assess the differences in the quantity and composition of CSHLPM in plain, hill, and mountainous areas, and Anhui Province of eastern China was used a typical research case. The results of the study indicated that the composition of agricultural and animal husbandry waste varied significantly across the three topographic areas. The content of nutrients (N, P_2_O_5_, and K_2_O) in these wastes was strongly correlated with the types of crop straw, livestock, and poultry. Among the three regions, the plain area was the highest producer of crop straw, followed by the hilly area, and the mountainous area was the lowest. In terms of regional distribution, the nutrients in CSHLPM were mainly concentrated in the plain area and hilly area. The proportion of nutrients in crop straw in the mountainous areas (N-50.8%, P_2_O_5_-50.0%, and K_2_O-78.4%) was higher than that in the other two regions (plain area: N-45.2%, P_2_O_5_-48.4%, and K_2_O-67.9%; hilly area: N-40.0%, P_2_O_5_-39.9%, and K_2_O-69.6%). In terms of nutrient sources, although the composition proportion of various annual average nutrients fell in the order of crop straw > feces > urine, the proportion of nutrients K_2_O from crop straw was the highest at 70%, while the proportion of nutrients-N and nutrients-P_2_O_5_ from crop straw was only 45.3% and 46.2%, respectively.

The potential of CSHLPM to contribute to agricultural and environmental benefits was evaluated by comparing its nutrient content to the nutrient demand of crop growth in historical periods. It was found that while CSHLPM can be used as a supplement, it should not completely replace the use of chemical fertilizer to meet nutrient needs, and K_2_O is the only nutrient that can be fully satisfied by reusing CSHLPM in all regions. However, in plain and hilly areas, the N and P_2_O_5_ requirements cannot be met by the CSHLPM alone. Notably, the annual average substitution sufficiency of N, P_2_O_5_ and K_2_O in hilly areas reached 100%, making it the only region where chemical fertilizer could be completely replaced by CSHLPM.

Finally, this study provides a detailed analysis of the characteristics of different topographic areas and offers two constructive suggestions for the management strategy of CSHLPM. These include adjusting the industrial structure of husbandry and planting and specifying recommended ratios for mixing organic and chemical fertilizers. The results of this study provide useful information for the reasonable planning of the industrial structure of husbandry and planting and shed light on the potential of CSHLPM as an organic fertilizer return to farmland, thus advancing the discussion on the rural environment and returning organic fertilizers to farmland policies.

## Author contribution statement

Lei Chen; Jingyu Zhang: Conceived and designed the experiments; Performed the experiments; Analyzed and interpreted the data; Wrote the paper.

Xiaolin Xia; Zhi Yang: Contributed reagents, materials, analysis tools or data.

Bangwen Wang; Changyu Long: Analyzed and interpreted the data.

## Data availability statement

All data that support the findings of this study are included within the article.

## Additional information

Supplementary content related to this article has been published online at [URL].

## Declaration of competing interest

The authors declare that they have no known competing financial interests or personal relationships that could have appeared to influence the work reported in this paper.
