# Supplementary material for: The potential capability of substituting chemical fertilizers with crop straw and human-livestock-poultry manure in areas with different topographic characteristics
Source: Heliyon. 2023 Aug 1;9(8):e18845. doi: 10.1016/j.heliyon.2023.e18845 (PMC10412849; doi:10.1016/j.heliyon.2023.e18845)
Supplement: Multimedia component 1 [file mmc1.docx]

Supplementary Materials for

**The Potential Capability for Substituting Chemical Fertilizer with Crop Straw and Human-Livestock-Poultry Manure in Areas with Different Topographic Characteristics**

**This file includes**

Tables S1–S5

Figure S1

**Table S1**

Crop sown areas of the 5 main crops across the 16 municipalities in Anhui province from 2009 to 2020. Unit: 10^3^ hectares

| Area | Municipalities | 2011 | 2012 | 2013 | 2014 | 2015 | 2016 | 2017 | 2018 | 2019 | 2020 |
| --- | --- | --- | --- | --- | --- | --- | --- | --- | --- | --- | --- |
| Plain area | Bengbu | 478.0 | 455.3 | 457.1 | 469.9 | 474.8 | 483.2 | 490.3 | 515.6 | 514.8 | 515.2 |
|  | Huaibei | 265.7 | 233.8 | 235.0 | 238.6 | 240.4 | 242.6 | 246.5 | 275.6 | 275.0 | 275.1 |
|  | Huainan | 212.0 | 203.1 | 204.0 | 207.0 | 432.4 | 439.8 | 435.0 | 531.9 | 526.9 | 526.3 |
|  | Fuyang | 1016.6 | 996.3 | 1000.0 | 1002.0 | 999.9 | 1003.2 | 1005.3 | 973.1 | 969.7 | 970.7 |
|  | Suzhou | 791.6 | 797.4 | 803.4 | 818.2 | 826.8 | 832.5 | 840.9 | 941.6 | 939.0 | 937.6 |
|  | Bozhou | 846.3 | 834.3 | 837.9 | 859.8 | 866.8 | 870.1 | 884.0 | 868.1 | 864.2 | 863.6 |
|  | sum | 3610.2 | 3520.3 | 3537.4 | 3595.5 | 3841.1 | 3871.6 | 3901.9 | 4105.9 | 4089.5 | 4088.4 |
| Hilly area | Hefei | 473.5 | 481.7 | 483.6 | 491.0 | 495.2 | 500.4 | 507.9 | 525.0 | 522.4 | 522.3 |
|  | Wuhu | 198.5 | 197.4 | 198.4 | 204.4 | 206.2 | 209.1 | 212.5 | 221.7 | 220.8 | 222.4 |
|  | Maanshan | 146.1 | 153.2 | 153.5 | 155.1 | 156.8 | 158.3 | 160.6 | 176.9 | 176.2 | 176.0 |
|  | Tongling | 27.0 | 27.8 | 28.0 | 28.6 | 126.1 | 127.8 | 125.3 | 101.1 | 100.7 | 100.4 |
|  | Chuzhou | 699.0 | 708.6 | 711.7 | 722.1 | 730.6 | 738.6 | 741.3 | 833.5 | 829.9 | 826.8 |
|  | sum | 1544.1 | 1568.7 | 1575.2 | 1601.2 | 1715.0 | 1734.2 | 1747.6 | 1858.3 | 1850.1 | 1847.8 |
| Mountainous area | Anqing | 450.4 | 454.1 | 455.7 | 460.0 | 363.2 | 367.8 | 365.1 | 345.5 | 344.0 | 344.9 |
|  | Huangshan | 65.0 | 64.8 | 64.4 | 64.3 | 62.4 | 61.7 | 59.4 | 50.7 | 50.7 | 51.0 |
|  | Lu’an | 701.2 | 715.4 | 718.8 | 730.0 | 507.8 | 515.2 | 509.7 | 610.1 | 607.4 | 608.8 |
|  | Chizhou | 114.1 | 115.4 | 115.9 | 117.4 | 118.7 | 119.4 | 119.3 | 118.0 | 117.6 | 118.6 |
|  | Xuancheng | 230.1 | 230.1 | 230.9 | 232.2 | 232.4 | 234.3 | 231.1 | 218.4 | 217.7 | 218.0 |
|  | sum | 1560.8 | 1579.8 | 1585.7 | 1603.8 | 1284.4 | 1298.4 | 1284.6 | 1342.7 | 1337.4 | 1341.3 |

**Table S2**

Grain outputs of the 5 main crops across the 16 municipalities in Anhui province from 2011 to 2020. Unit: million tons

| Area | Municipalities | 2011 | 2012 | 2013 | 2014 | 2015 | 2016 | 2017 | 2018 | 2019 | 2020 |
| --- | --- | --- | --- | --- | --- | --- | --- | --- | --- | --- | --- |
| Plain area | Bengbu | 2.8 | 2.6 | 2.6 | 2.8 | 2.9 | 2.8 | 2.9 | 2.8 | 2.8 | 2.8 |
|  | Huaibei | 1.3 | 1.2 | 1.2 | 1.2 | 1.3 | 1.3 | 1.3 | 1.4 | 1.5 | 1.5 |
|  | Huainan | 1.4 | 1.3 | 1.3 | 1.4 | 2.9 | 2.8 | 2.9 | 3.2 | 3.2 | 3.1 |
|  | Fuyang | 5.4 | 5.2 | 5.3 | 5.5 | 5.7 | 5.6 | 5.7 | 5.1 | 5.2 | 5.2 |
|  | Suzhou | 4.0 | 3.8 | 3.8 | 4.0 | 4.1 | 4.0 | 4.1 | 4.3 | 4.4 | 4.5 |
|  | Bozhou | 4.7 | 4.4 | 4.4 | 4.5 | 4.7 | 4.6 | 4.7 | 4.8 | 5.0 | 5.0 |
|  | sum | 19.5 | 18.5 | 18.6 | 19.4 | 21.6 | 21.1 | 21.5 | 21.7 | 22.1 | 22.1 |
| Hilly area | Hefei | 3.2 | 3.0 | 3.0 | 3.1 | 3.2 | 3.1 | 3.2 | 3.0 | 3.0 | 2.9 |
|  | Wuhu | 1.4 | 1.3 | 1.3 | 1.4 | 1.4 | 1.4 | 1.4 | 1.4 | 1.4 | 1.4 |
|  | Maanshan | 1.1 | 1.0 | 1.0 | 1.1 | 1.1 | 1.1 | 1.1 | 1.1 | 1.1 | 1.0 |
|  | Tongling | 0.2 | 0.2 | 0.2 | 0.2 | 0.7 | 0.6 | 0.7 | 0.6 | 0.6 | 0.6 |
|  | Chuzhou | 4.4 | 4.1 | 4.1 | 4.3 | 4.4 | 4.3 | 4.4 | 4.6 | 4.6 | 4.7 |
|  | sum | 10.3 | 9.7 | 9.6 | 10.0 | 10.9 | 10.4 | 10.6 | 10.7 | 10.7 | 10.5 |
| Mountainous area | Anqing | 2.7 | 2.5 | 2.5 | 2.6 | 2.2 | 2.1 | 2.1 | 2.0 | 1.9 | 1.9 |
|  | Huangshan | 0.4 | 0.3 | 0.3 | 0.3 | 0.3 | 0.3 | 0.3 | 0.3 | 0.3 | 0.3 |
|  | Lu’an | 4.8 | 4.5 | 4.4 | 4.6 | 3.3 | 3.1 | 3.2 | 3.5 | 3.5 | 3.5 |
|  | Chizhou | 0.7 | 0.7 | 0.7 | 0.7 | 0.7 | 0.7 | 0.7 | 0.6 | 0.6 | 0.6 |
|  | Xuancheng | 1.4 | 1.3 | 1.3 | 1.3 | 1.4 | 1.3 | 1.3 | 1.3 | 1.3 | 1.3 |
|  | sum | 9.9 | 9.3 | 9.2 | 9.6 | 7.9 | 7.5 | 7.6 | 7.7 | 7.6 | 7.5 |

**Table S3**

The number of livestock, poultry, and humans from 2011 to 2020. Unit: millions

| Area | Species | 2011 | 2012 | 2013 | 2014 | 2015 | 2016 | 2017 | 2018 | 2019 | 2020 |
| --- | --- | --- | --- | --- | --- | --- | --- | --- | --- | --- | --- |
| Plain area | Human | 15.504 | 15.125 | 14.928 | 14.766 | 15.189 | 14.972 | 14.727 | 14.487 | 14.217 | 13.999 |
|  | Cattle | 0.876 | 0.914 | 0.949 | 0.959 | 1.032 | 1.058 | 1.061 | 0.581 | 0.577 | 0.663 |
|  | Sheep | 5.551 | 5.824 | 6.059 | 6.259 | 6.923 | 7.195 | 7.366 | 4.408 | 4.775 | 5.107 |
|  | Poultry | 97.452 | 104.050 | 111.869 | 114.147 | 136.362 | 139.296 | 141.807 | 95.694 | 109.560 | 122.014 |
|  | Pig | 23.180 | 24.275 | 24.873 | 25.712 | 27.487 | 27.327 | 27.532 | 21.253 | 19.453 | 20.328 |
| Hilly area | Human | 10.862 | 10.517 | 10.255 | 10.039 | 9.244 | 8.982 | 8.703 | 8.583 | 8.400 | 7.068 |
|  | Cattle | 0.530 | 0.526 | 0.527 | 0.527 | 0.434 | 0.401 | 0.377 | 0.140 | 0.145 | 0.168 |
|  | Sheep | 0.896 | 0.938 | 0.990 | 1.027 | 0.864 | 0.913 | 0.903 | 0.583 | 0.643 | 0.658 |
|  | Poultry | 133.883 | 147.148 | 146.275 | 148.354 | 140.649 | 139.083 | 139.647 | 80.272 | 93.948 | 103.494 |
|  | Pig | 16.164 | 16.794 | 17.180 | 17.576 | 16.138 | 15.925 | 16.036 | 14.190 | 11.225 | 9.622 |
| Mountainous area | Human | 6.404 | 6.247 | 6.113 | 6.006 | 5.927 | 5.799 | 5.660 | 5.645 | 5.572 | 4.374 |
|  | Cattle | 0.234 | 0.227 | 0.227 | 0.215 | 0.210 | 0.206 | 0.204 | 0.101 | 0.105 | 0.119 |
|  | Sheep | 0.101 | 0.117 | 0.135 | 0.146 | 0.171 | 0.195 | 0.200 | 0.137 | 0.150 | 0.215 |
|  | Poultry | 62.947 | 67.902 | 75.049 | 74.785 | 78.717 | 75.801 | 74.858 | 58.674 | 79.570 | 84.734 |
|  | Pig | 8.522 | 8.882 | 9.041 | 9.192 | 9.212 | 8.940 | 8.875 | 7.287 | 5.677 | 5.750 |

**Table S4**

Quantity of excrement resources from livestock, poultry, and humans from 2011 to 2020. Unit: million tons

| Area | Types | Wastes | 2011 | 2012 | 2013 | 2014 | 2015 | 2016 | 2017 | 2018 | 2019 | 2020 |
| --- | --- | --- | --- | --- | --- | --- | --- | --- | --- | --- | --- | --- |
| Plain area | Human | Faeces | 0.85 | 0.83 | 0.82 | 0.81 | 0.83 | 0.82 | 0.81 | 0.79 | 0.78 | 0.77 |
|  |  | Urine | 11.32 | 11.04 | 10.90 | 10.78 | 11.09 | 10.93 | 10.75 | 10.58 | 10.38 | 10.22 |
|  | Cattle | Faeces | 6.40 | 6.67 | 6.93 | 7.00 | 7.54 | 7.72 | 7.74 | 4.24 | 4.21 | 4.84 |
|  |  | Urine | 3.20 | 3.34 | 3.47 | 3.50 | 3.77 | 3.86 | 3.87 | 2.12 | 2.11 | 2.42 |
|  | Sheep | Faeces | 5.27 | 5.53 | 5.75 | 5.94 | 6.57 | 6.83 | 6.99 | 4.18 | 4.53 | 4.85 |
|  |  | Urine | 1.30 | 1.36 | 1.42 | 1.46 | 1.62 | 1.68 | 1.72 | 1.03 | 1.12 | 1.19 |
|  | Poultry | Faeces | 2.46 | 2.62 | 2.82 | 2.88 | 3.44 | 3.51 | 3.57 | 2.41 | 2.76 | 3.07 |
|  |  | Urine | - | - | - | - | - | - | - | - | - | - |
|  | Pig | Faeces | 9.23 | 9.66 | 9.90 | 10.23 | 10.94 | 10.88 | 10.96 | 8.46 | 7.74 | 8.09 |
|  |  | Urine | 15.22 | 15.94 | 16.33 | 16.89 | 18.05 | 17.95 | 18.08 | 13.96 | 12.77 | 13.35 |
| Hilly area | Human | Faeces | 0.59 | 0.58 | 0.56 | 0.55 | 0.51 | 0.49 | 0.48 | 0.47 | 0.46 | 0.39 |
|  |  | Urine | 7.93 | 7.68 | 7.49 | 7.33 | 6.75 | 6.56 | 6.35 | 6.27 | 6.13 | 5.16 |
|  | Cattle | Faeces | 3.87 | 3.84 | 3.85 | 3.85 | 3.17 | 2.93 | 2.75 | 1.02 | 1.06 | 1.23 |
|  |  | Urine | 1.94 | 1.92 | 1.92 | 1.92 | 1.58 | 1.46 | 1.38 | 0.51 | 0.53 | 0.61 |
|  | Sheep | Faeces | 0.85 | 0.89 | 0.94 | 0.98 | 0.82 | 0.87 | 0.86 | 0.55 | 0.61 | 0.62 |
|  |  | Urine | 0.21 | 0.22 | 0.23 | 0.24 | 0.20 | 0.21 | 0.21 | 0.14 | 0.15 | 0.15 |
|  | Poultry | Faeces | 3.37 | 3.71 | 3.69 | 3.74 | 3.54 | 3.50 | 3.52 | 2.02 | 2.37 | 2.61 |
|  |  | Urine | - | - | - | - | - | - | - | - | - | - |
|  | Pig | Faeces | 6.43 | 6.68 | 6.84 | 7.00 | 6.42 | 6.34 | 6.38 | 5.65 | 4.47 | 3.83 |
|  |  | Urine | 10.61 | 11.03 | 11.28 | 11.54 | 10.60 | 10.46 | 10.53 | 9.32 | 7.37 | 6.32 |
| Mountainous area | Human | Faeces | 0.35 | 0.34 | 0.33 | 0.33 | 0.32 | 0.32 | 0.31 | 0.31 | 0.31 | 0.24 |
|  |  | Urine | 4.68 | 4.56 | 4.46 | 4.38 | 4.33 | 4.23 | 4.13 | 4.12 | 4.07 | 3.19 |
|  | Cattle | Faeces | 1.71 | 1.65 | 1.66 | 1.57 | 1.54 | 1.50 | 1.49 | 0.74 | 0.77 | 0.87 |
|  |  | Urine | 0.85 | 0.83 | 0.83 | 0.79 | 0.77 | 0.75 | 0.74 | 0.37 | 0.38 | 0.43 |
|  | Sheep | Faeces | 0.10 | 0.11 | 0.13 | 0.14 | 0.16 | 0.19 | 0.19 | 0.13 | 0.14 | 0.20 |
|  |  | Urine | 0.02 | 0.03 | 0.03 | 0.03 | 0.04 | 0.05 | 0.05 | 0.03 | 0.03 | 0.05 |
|  | Poultry | Faeces | 1.59 | 1.71 | 1.89 | 1.88 | 1.98 | 1.91 | 1.89 | 1.48 | 2.01 | 2.14 |
|  |  | Urine | - | - | - | - | - | - | - | - | - | - |
|  | Pig | Faeces | 3.39 | 3.53 | 3.60 | 3.66 | 3.67 | 3.56 | 3.53 | 2.90 | 2.26 | 2.29 |
|  |  | Urine | 5.60 | 5.83 | 5.94 | 6.04 | 6.05 | 5.87 | 5.83 | 4.79 | 3.73 | 3.78 |

**Table S5**

The potential capability for substituting chemical fertilizer with CSHLPM

| **Areas** | **Nutrient** | **Whether or not** | **The degree of replacement** |
| --- | --- | --- | --- |
| **Plain area** | **N** | Partial substitution | 93.9% |
|  | **P_2_O_5_** | Partial substitution | 80.0% |
|  | **K_2_O** | Completely replace | 100% |
| **Hilly area** | **N** | Completely replace | 100% |
|  | **P_2_O_5_** | Completely replace | 100% |
|  | **K_2_O** | Completely replace | 100% |
| **Mountainous area** | **N** | Completely replace | 100% |
|  | **P_2_O_5_** | Partial substitution | 88.6% |
|  | **K_2_O** | Completely replace | 100% |


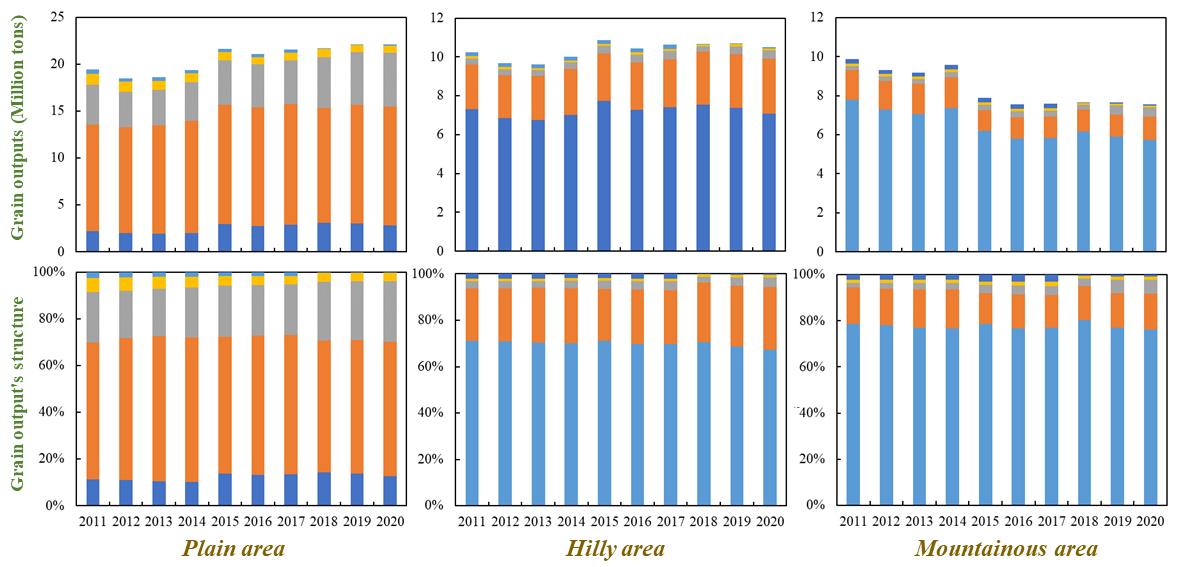


**Figure S1**

Quantity and composition of crops in the three topographic areas from 2011 to 2020.
